# Body roundness index and self-reported oral health among US adults: Nonlinear patterns and an exploratory indirect association through the systemic immune-inflammation index

**DOI:** 10.1097/MD.0000000000049981

**Published:** 2026-07-24

**Authors:** Jilun Liu, Wei Yang, Shuning Li

**Affiliations:** a Department of Oral and Maxillofacial Surgery, The Second Hospital of Hebei Medical University, Shijiazhuang, Hebei, China.

**Keywords:** body roundness index, central adiposity, National Health and Nutrition Examination Survey, self-reported oral health, systemic immune-inflammation index

## Abstract

Evidence linking body roundness index (BRI) to self-reported oral health is limited. We examined this association, nonlinearity, subgroup differences, and an indirect association through the systemic immune-inflammation index (SII). This cross-sectional study included 14,656 adults from the 2009 to 2014 National Health and Nutrition Examination Survey. Self-reported oral health was analyzed as a 5-level ordered outcome. Survey-weighted ordinal logistic regression, restricted cubic spline, fixed-folding point, subgroup, and threshold-specific sensitivity analyses were performed. Exploratory mediation analysis used 1000 bootstrap simulations. In the fully adjusted model, each 1-unit increase in BRI was associated with higher cumulative odds of poorer self-reported oral health (odds ratio [OR], 1.076; 95% confidence interval [CI], 1.055–1.097). Compared with the lowest BRI tertile, the OR for the highest tertile was 1.448 (95% CI, 1.272–1.647). The spline showed modest nonlinearity (*P* = .048). Below the fixed-folding-point at BRI = 10, the OR was 1.092 (95% CI, 1.063–1.120); above 10, the association was attenuated (OR, 0.978; 95% CI, 0.913–1.048). Although the proportional-odds assumption was not fully supported (*P* = .011), threshold-specific ORs remained positive (1.065–1.099). Interactions were significant for age and education level. SII accounted for 3.06% (95% CI, 0.99%–5.82%) of the association. Higher BRI was associated with poorer self-reported oral health among US adults. The association varied modestly across thresholds and attenuated at higher BRI levels, while SII explained only a small proportion. Prospective studies with clinical dental assessments are needed.

## 1. Introduction

Obesity is a major global public health concern and is closely associated with cardiovascular disease, type 2 diabetes, certain cancers, musculoskeletal disorders, and premature mortality.^[1–3]^ In 2022, approximately 2.5 billion adults worldwide were overweight, including more than 890 million living with obesity. Conventional assessments of obesity have predominantly relied on body mass index (BMI).^[4,5]^ However, BMI does not adequately distinguish fat mass from lean mass or characterize the distribution of adipose tissue.^[6]^ Central adiposity may be particularly relevant because abdominal fat accumulation is strongly associated with metabolic dysfunction and chronic low-grade inflammation.^[6–8]^ The body roundness index (BRI) is a geometrically derived anthropometric measure calculated from waist circumference (WC) and height.^[9,10]^ By quantifying body shape independently of body weight, BRI may better reflect central body configuration and visceral adiposity than BMI, while remaining inexpensive and readily applicable in population-based studies.^[10,11]^

Oral health is an essential component of general health and contributes to nutrition, communication, social functioning, and quality of life.^[12–14]^ Nevertheless, oral diseases remain among the most prevalent noncommunicable diseases worldwide. Approximately 3.5 billion people are affected by oral diseases globally, including an estimated 2.5 billion with untreated dental caries, 1 billion with severe periodontal disease, and 350 million with complete tooth loss.^[15–17]^ These conditions share several risk factors and determinants with obesity, including unhealthy dietary patterns, tobacco use, socioeconomic disadvantage, limited access to healthcare, metabolic abnormalities, and systemic inflammation.^[18–20]^ Self-reported oral health provides a concise, patient-centered assessment of the perceived condition of the teeth and gums and may capture symptoms, functional limitations, and healthcare needs that are not fully represented by any single clinical measure.^[21–23]^

Although obesity has been linked to adverse oral-health outcomes, evidence regarding the relationship between BRI and self-reported oral health remains scarce. It also remains unclear whether this association differs across population subgroups or across different levels of self-reported oral health. Therefore, using nationally representative data from the National Health and Nutrition Examination Survey (NHANES) 2009 to 2014, we examined the association between BRI and self-reported oral health while accounting for the complex survey design.

## 2. Materials and methods

### 2.1. Study design and population

This cross-sectional study used data from 3 consecutive NHANES cycles: 2009 to 2010, 2011 to 2012, and 2013 to 2014. NHANES is conducted by the National Center for Health Statistics and uses a complex, multistage probability sampling design to obtain a nationally representative sample of the noninstitutionalized civilian population of the United States. The NHANES protocols were approved by the National Center for Health Statistics Research Ethics Review Board, and written informed consent was obtained from all participants.^[24]^

**Figure 1.**
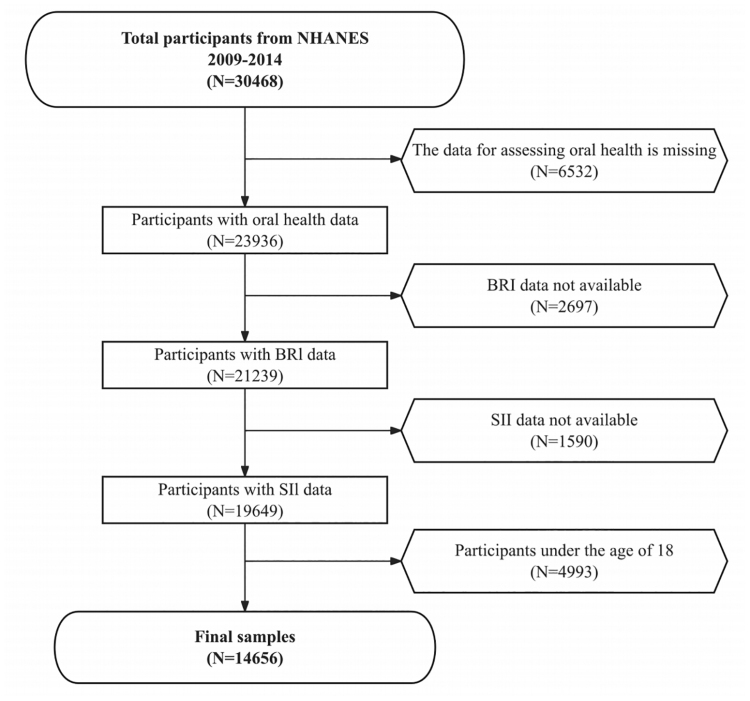
Flowchart of participant selection. NHANES = National Health and Nutrition Examination Survey; BRI = body roundness index, SII = systemic immune-inflammation index.

A total of 30,468 participants were initially identified. Participants were excluded if information on self-reported oral health was unavailable (n = 6532), BRI could not be calculated (n = 2697), systemic immune-inflammation index (SII) data were unavailable (n = 1590), or they were younger than 18 years (n = 4993). The final analytical sample comprised 14,656 adults (Fig. 1).

### 2.2. Assessment of self-reported oral health

Self-reported oral health was assessed using item OHQ845 of the NHANES Oral Health Questionnaire: “Overall, how would you rate the health of your teeth and gums?” The response options were excellent, very good, good, fair, and poor. Responses were coded from 1 to 5 and analyzed as a 5-level ordered outcome, with higher categories indicating poorer self-reported oral health.

### 2.3. BRI and SII

BRI was calculated from standing height (BMXHT) and WC (BMXWAIST) using the equation BRI = 364.2 − 365.5 × √(1 − [(WC/(2π))^2^/(0.5 × height)^2^]),^[25]^ with WC and height expressed in the same unit. BRI was examined both continuously and categorically using survey-weighted tertiles. The weighted cut points were 4.124 and 5.887, defining Q1 (BRI ≤ 4.124), Q2 (4.124 < BRI ≤ 5.887), and Q3 (BRI > 5.887).

SII was calculated as platelet count × neutrophil count/lymphocyte count, using platelet count (LBXPLTSI), neutrophil count (LBDNENO), and lymphocyte count (LBDLYMNO).^[26]^

### 2.4. Covariates

Covariates were selected on the basis of their potential relationships with adiposity and oral health. Demographic and socioeconomic covariates included gender (RIAGENDR), age (RIDAGEYR), race/ethnicity (RIDRETH1), education level (DMDEDUC2), and the ratio of family income to poverty (RIP; INDFMPIR). Age was categorized as <65 or ≥65 years in the primary regression models. Race/ethnicity was classified as Mexican American, other Hispanic, non-Hispanic White, non-Hispanic Black, or other race. Education level was classified as <9th grade, 9th to 11th grade, high school graduate/General Educational Development or equivalent, some college or associate degree, or college graduate or above. RIP was modeled as a continuous variable.

Clinical covariates included diabetes status (DIQ010), hypertension (BPQ020), coronary heart disease (MCQ160C), hypercholesterolemia (BPQ080), arthritis (MCQ160A), stroke (MCQ160F), and cancer or malignancy (MCQ220). Cigarette use was classified as never, former, or current smoking, and alcohol use was classified as never, moderate, or heavy drinking.^[27]^ Detailed calculation methods and classification criteria are provided in Table S1, Supplemental Digital Content 1.

### 2.5. Missing data

Missing covariate data were present for education level (0.09%), RIP (7.29%), diabetes (0.05%), hypertension (0.10%), coronary heart disease (0.29%), hypercholesterolemia (0.10%), arthritis (0.21%), stroke (0.07%), cancer or malignancy (0.07%), cigarette use (0.03%), and alcohol use (22.60%). Gender, age, race/ethnicity, self-reported oral health, BRI, and SII had no missing values in the analytical sample. Missing covariate values were imputed using a random forest-based algorithm before model fitting. A single completed dataset was generated and used for all subsequent analyses.

### 2.6. Complex survey design

All descriptive analyses and the primary ordinal regression, restricted cubic spline, threshold, subgroup, proportional-odds diagnostic, and threshold-specific sensitivity analyses accounted for the NHANES complex sampling design. Because three 2-year survey cycles were combined, the final examination weight was calculated as WTMEC2YR/3. Survey design objects incorporated the primary sampling unit (SDMVPSU), stratum variable (SDMVSTRA), and the combined examination weight.

### 2.7. Statistical analysis

Survey-weighted continuous variables are presented as weighted means with standard deviations, and categorical variables are presented as weighted population counts and percentages. Baseline characteristics were compared across BRI tertiles using Rao–Scott adjusted chi-square tests for categorical variables and design-based Kruskal–Wallis tests for continuous variables. Corresponding unweighted baseline characteristics are presented in Table S2, Supplemental Digital Content 2.

Survey-weighted ordinal logistic regression was used to evaluate the association between BRI and self-reported oral health. Results are reported as common cumulative odds ratios (ORs) with 95% confidence intervals (CIs). An OR > 1 indicates higher cumulative odds of being in a poorer self-reported oral-health category. BRI was modeled continuously and by survey-weighted tertiles, with Q1 as the reference group. Model 1 was unadjusted. Model 2 was adjusted for gender, age, race/ethnicity, education level, and RIP. Model 3 was additionally adjusted for diabetes, hypertension, coronary heart disease, hypercholesterolemia, arthritis, stroke, cancer or malignancy, cigarette use, and alcohol use. Linear trends across BRI tertiles were evaluated by entering the ordered tertile variable as a continuous term.

The proportional-odds assumption for the primary exposure was evaluated by fitting 4 survey-weighted binary logistic regression models corresponding to the cumulative outcome thresholds. Differences in the BRI coefficient across thresholds were formally assessed in a stacked survey-weighted logistic model containing a threshold-by-BRI interaction term, using a design-based Wald test. Because the assumption was not fully supported, threshold-specific ORs were reported as a sensitivity analysis (Table S3, Supplemental Digital Content 3), and the common cumulative ORs from the ordinal models were interpreted as overall summary estimates.

Subgroup analyses were performed across demographic, socioeconomic, clinical, and lifestyle strata. Potential effect modification was evaluated by including multiplicative interaction terms between BRI and the corresponding stratification variable (Fig. S1, Supplemental Digital Content 4).

An exploratory mediation analysis was conducted using the R package mediation to assess the indirect statistical association through SII. For this analysis only, self-reported oral health was represented as a numerical score ranging from 1 (excellent) to 5 (poor), with higher scores indicating poorer self-reported oral health. Ordinary linear regression was used for both the mediator model, in which SII was the dependent variable, and the outcome model, in which the oral-health score was the dependent variable and both BRI and SII were included as predictors. Total, direct, and indirect effects represent the mean change in the 1-to-5 oral-health score associated with a 1-unit increase in BRI. Covariates were entered according to the Model 2 and Model 3 adjustment sets, and 95% CIs were estimated using 1000 nonparametric bootstrap simulations. The mediation analysis was treated as exploratory and was not used to support causal inference.

All analyses were performed using R version 4.2.1 (R Foundation for Statistical Computing, Vienna, Austria). Statistical tests were two-sided, and *P* < .05 was considered statistically significant.

## 3. Results

### 3.1. Study population and baseline characteristics

The final analysis included 14,656 participants, representing a survey-weighted population of 195,776,703 US adults. The weighted population totals were 65,287,753 in Q1, 65,256,640 in Q2, and 65,232,310 in Q3. Overall, 48.6% were male, 82.6% were younger than 65 years, and 67.8% were non-Hispanic White. Self-reported oral health was rated as excellent by 12.7%, very good by 24.2%, good by 36.1%, fair by 18.3%, and poor by 8.7% (Table 1).

**Table 1:** Characterization of the study population.

Characteristic	Overall Weighted N = 195,776,703*	Q1 Weighted N = 65,287,753*	Q2 Weighted N = 65,256,640*	Q3 Weighted N = 65,232,310*	*P*-value†
Gender					<.001
Male	95,109,410 (48.6%)	33,091,143 (50.7%)	34,954,952 (53.6%)	27,063,314 (41.5%)	
Female	100,667,293 (51.4%)	32,196,610 (49.3%)	30,301,688 (46.4%)	38,168,996 (58.5%)	
Age, yr					<.001
<65	161,650,767 (82.6%)	59,143,998 (90.6%)	52,685,692 (80.7%)	49,821,077 (76.4%)	
≥65	34,125,936 (17.4%)	6,143,755 (9.4%)	12,570,948 (19.3%)	15,411,233 (23.6%)	
Race					<.001
Mexican American	16,408,056 (8.4%)	3,508,524 (5.4%)	6,137,587 (9.4%)	6,761,944 (10.4%)	
Other Hispanic	11,388,342 (5.8%)	3,376,116 (5.2%)	4,019,326 (6.2%)	3,992,899 (6.1%)	
Non-Hispanic White	132,700,797 (67.8%)	44,904,289 (68.8%)	44,205,064 (67.7%)	43,591,443 (66.8%)	
Non-Hispanic Black	21,012,160 (10.7%)	6,702,743 (10.3%)	6,166,873 (9.5%)	8,142,544 (12.5%)	
Other race	14,267,349 (7.3%)	6,796,079 (10.4%)	4,727,789 (7.2%)	2,743,480 (4.2%)	
Education level					<.001
<9th grade	10,091,885 (5.2%)	1,732,633 (2.7%)	3,728,155 (5.7%)	4,631,098 (7.1%)	
9–11th grade	20,847,600 (10.6%)	5,495,743 (8.4%)	7,009,770 (10.7%)	8,342,086 (12.8%)	
High school Grad/GED or equivalent	42,647,590 (21.8%)	12,362,682 (18.9%)	13,951,569 (21.4%)	16,333,339 (25.0%)	
Some college or AA degree	62,566,111 (32.0%)	19,864,511 (30.4%)	20,575,309 (31.5%)	22,126,291 (33.9%)	
College graduate or above	59,623,518 (30.5%)	25,832,184 (39.6%)	19,991,838 (30.6%)	13,799,496 (21.2%)	
RIP	2.94 (1.63)	3.07 (1.66)	3.04 (1.62)	2.71 (1.58)	<.001
Diabetes					<.001
Yes	17,851,198 (9.1%)	1,535,480 (2.4%)	4,396,167 (6.7%)	11,919,551 (18.3%)	
No	173,458,424 (88.6%)	63,208,544 (96.8%)	59,261,150 (90.8%)	50,988,729 (78.2%)	
Borderline	4,467,081 (2.3%)	543,728 (0.8%)	1,599,323 (2.5%)	2,324,030 (3.6%)	
Hypertension					<.001
Yes	63,055,719 (32.2%)	9,523,013 (14.6%)	21,181,540 (32.5%)	32,351,166 (49.6%)	
No	132,720,984 (67.8%)	55,764,739 (85.4%)	44,075,100 (67.5%)	32,881,145 (50.4%)	
Coronary heart disease					<.001
Yes	6,380,177 (3.3%)	891,623 (1.4%)	2,291,322 (3.5%)	3,197,233 (4.9%)	
No	189,396,526 (96.7%)	64,396,130 (98.6%)	62,965,319 (96.5%)	62,035,077 (95.1%)	
Hypercholesterolemia					<.001
Yes	66,522,581 (34.0%)	12,894,642 (19.8%)	25,091,623 (38.5%)	28,536,315 (43.7%)	
No	129,254,122 (66.0%)	52,393,110 (80.2%)	40,165,017 (61.5%)	36,695,995 (56.3%)	
Arthritis					<.001
Yes	48,305,548 (24.7%)	9,053,176 (13.9%)	15,469,524 (23.7%)	23,782,848 (36.5%)	
No	147,471,155 (75.3%)	56,234,576 (86.1%)	49,787,117 (76.3%)	41,449,462 (63.5%)	
Stroke					<.001
Yes	4,811,418 (2.5%)	1,127,054 (1.7%)	1,393,366 (2.1%)	2,290,998 (3.5%)	
No	190,965,285 (97.5%)	64,160,698 (98.3%)	63,863,275 (97.9%)	62,941,312 (96.5%)	
Cancer or malignancy					<.001
Yes	19,603,079 (10.0%)	4,904,021 (7.5%)	6,719,467 (10.3%)	7,979,591 (12.2%)	
No	176,173,624 (90.0%)	60,383,731 (92.5%)	58,537,173 (89.7%)	57,252,719 (87.8%)	
Cigarette use					<.001
Never smoker	109,267,763 (55.8%)	37,883,261 (58.0%)	36,337,909 (55.7%)	35,046,593 (53.7%)	
Former smoker	49,376,301 (25.2%)	13,247,502 (20.3%)	17,156,652 (26.3%)	18,972,146 (29.1%)	
Current smoker	37,132,639 (19.0%)	14,156,989 (21.7%)	11,762,079 (18.0%)	11,213,571 (17.2%)	
Alcohol use					.001
Never drinking	21,046,046 (10.8%)	5,987,538 (9.2%)	6,661,418 (10.2%)	8,397,089 (12.9%)	
Moderate drinking	167,838,137 (85.7%)	56,999,424 (87.3%)	56,207,599 (86.1%)	54,631,114 (83.7%)	
Heavy drinking	6,892,521 (3.5%)	2,300,791 (3.5%)	2,387,623 (3.7%)	2,204,106 (3.4%)	
Self-reported oral health					<.001
Excellent	24,823,185 (12.7%)	9,510,367 (14.6%)	8,339,534 (12.8%)	6,973,285 (10.7%)	
Very good	47,475,693 (24.2%)	18,832,666 (28.8%)	15,699,448 (24.1%)	12,943,579 (19.8%)	
Good	70,700,233 (36.1%)	22,679,988 (34.7%)	24,328,598 (37.3%)	23,691,646 (36.3%)	
Fair	35,767,793 (18.3%)	10,170,885 (15.6%)	11,840,787 (18.1%)	13,756,121 (21.1%)	
Poor	17,009,799 (8.7%)	4,093,846 (6.3%)	5,048,274 (7.7%)	7,867,679 (12.1%)	

Values are survey-weighted N (%) or survey-weighted mean (SD). *P*-values were calculated using Rao–Scott adjusted chi-square tests or design-based Kruskal–Wallis tests.

GED = General Educational Development, RIP = ratio of family income to poverty.

* N (%); mean (SD).

† Pearson’s χ^2^: Rao & Scott adjustment; design-based Kruskal–Wallis test.

The distribution of self-reported oral health shifted progressively toward poorer categories with increasing BRI tertile. The weighted prevalence of fair oral health increased from 15.6% in Q1 to 21.1% in Q3, while the prevalence of poor oral health increased from 6.3% to 12.1%. Participants in higher BRI tertiles were also more likely to be aged ≥65 years and have diabetes, hypertension, coronary heart disease, hypercholesterolemia, arthritis, stroke, and cancer or malignancy. Most baseline characteristics differed significantly across BRI tertiles (*P* < .001), and alcohol-use patterns also differed across tertiles (*P* = .001). Corresponding unweighted characteristics are presented in Table S2, Supplemental Digital Content 2.

### 3.2. Survey-weighted univariable analyses

Survey-weighted univariable ordinal logistic regression results are presented in Table 2. Several demographic, socioeconomic, clinical, and behavioral factors were associated with self-reported oral health. Compared with men, women had lower cumulative odds of being in a poorer oral-health category (OR, 0.85; 95% CI, 0.80–0.90), and participants aged ≥65 years had lower cumulative odds than those aged <65 years (OR, 0.77; 95% CI, 0.69–0.86). Higher education and higher RIP were also associated with lower cumulative odds of poorer self-reported oral health.

**Table 2:** Survey-weighted univariable ordinal logistic regression analyses of factors associated with self-reported oral health.

	Weighted mean ± SD/weighted N (%)	OR (95% CI)	*P*-value
Gender			
Male	95,109,410 (48.58%)	Ref	
Female	100,667,293 (51.42%)	0.85 (0.80, 0.90)	<.0001
Age, yr			
<65	161,650,767 (82.57%)	Ref	
≥65	34,125,936 (17.43%)	0.77 (0.69, 0.86)	<.0001
Race			
Mexican American	16,408,056 (8.38%)	Ref	
Other Hispanic	11,388,342 (5.82%)	0.69 (0.58, 0.82)	<.0001
Non-Hispanic White	132,700,797 (67.78%)	0.35 (0.31, 0.40)	<.0001
Non-Hispanic Black	21,012,160 (10.73%)	0.65 (0.57, 0.74)	<.0001
Other race	14,267,349 (7.29%)	0.48 (0.43, 0.55)	<.0001
Education level			
<9th grade	10,091,885 (5.15%)	Ref	
9–11th grade	20,847,600 (10.65%)	0.84 (0.70, 1.00)	.0525
High school Grad/GED or equivalent	42,647,590 (21.78%)	0.53 (0.46, 0.62)	<.0001
Some college or AA degree	62,566,111 (31.96%)	0.36 (0.30, 0.44)	<.0001
College graduate or above	59,623,518 (30.45%)	0.17 (0.14, 0.19)	<.0001
RIP	2.94 ± 1.63	0.70 (0.68, 0.72)	<.0001
Diabetes			
Yes	17,851,198 (9.12%)	Ref	
No	173,458,424 (88.60%)	0.64 (0.58, 0.71)	<.0001
Borderline	4,467,081 (2.28%)	0.82 (0.65, 1.05)	.1156
Hypertension			
Yes	63,055,719 (32.21%)	Ref	
No	132,720,984 (67.79%)	0.80 (0.73, 0.88)	<.0001
Coronary heart disease			
Yes	6,380,177 (3.26%)	Ref	
No	189,396,526 (96.74%)	0.63 (0.51, 0.79)	.0001
Hypercholesterolemia			
Yes	66,522,581 (33.98%)	Ref	
No	129,254,122 (66.02%)	1.04 (0.92, 1.17)	.5224
Arthritis			
Yes	48,305,548 (24.67%)	Ref	
No	147,471,155 (75.33%)	0.85 (0.77, 0.93)	.0009
Stroke			
Yes	4,811,418 (2.46%)	Ref	
No	190,965,285 (97.54%)	0.78 (0.63, 0.95)	.0158
Cancer or malignancy			
Yes	19,603,079 (10.01%)	Ref	
No	176,173,624 (89.99%)	1.32 (1.16, 1.51)	<.0001
Cigarette use			
Never smoker	109,267,763 (55.81%)	Ref	
Former smoker	49,376,301 (25.22%)	1.26 (1.14, 1.39)	<.0001
Current smoker	37,132,639 (18.97%)	3.11 (2.74, 3.52)	<.0001
Alcohol use			
Never drinking	21,046,046 (10.75%)	Ref	
Moderate drinking	167,838,137 (85.73%)	1.04 (0.91, 1.19)	.5600
Heavy drinking	6,892,521 (3.52%)	0.91 (0.70, 1.17)	.4527

Descriptive values are presented as survey-weighted mean (SD) or survey-weighted N (%). Regression results are presented as survey-weighted cumulative ORs with 95% CIs and *P*-values, accounting for sampling weights, strata, and primary sampling units. An OR >1 indicates higher cumulative odds of being in a poorer self-reported oral-health category. For continuous variables, the OR corresponds to a 1-unit increase; for categorical variables, the first category is the reference.

CI = confidence interval, GED = General Educational Development, OR = odds ratio, RIP = ratio of family income to poverty.

### 3.3. Association between BRI and self-reported oral health

BRI was positively associated with poorer self-reported oral health in all 3 survey-weighted ordinal logistic models (Table 3). In Model 1, each 1-unit increase in BRI was associated with 11.1% higher common cumulative odds of being in a poorer oral-health category (OR, 1.111; 95% CI, 1.090–1.131; *P* < .0001). The association was attenuated but remained statistically significant after adjustment in Model 2 (OR, 1.085; 95% CI, 1.065–1.106; *P* < .0001) and Model 3 (OR, 1.076; 95% CI, 1.055–1.097; *P* < .0001).

**Table 3:** Association between BRI and self-reported oral health across different models.

Exposure	Model 1	Model 2	Model 3
BRI	1.111 (1.090, 1.131), <.0001	1.085 (1.065, 1.106), <.0001	1.076 (1.055, 1.097), <.0001
BRI tertiles			
Q1	Reference	Reference	Reference
Q2	1.262 (1.117, 1.425), .0004	1.206 (1.073, 1.355), .0025	1.188 (1.055, 1.337), .0064
Q3	1.737 (1.526, 1.978), <.0001	1.526 (1.339, 1.740), <.0001	1.448 (1.272, 1.647), <.0001
*P* for trend	<.0001	<.0001	<.0001

Results in table: OR (95% CI), *P*-value.

Outcome variable: self-reported oral health.

Exposure variable: BRI.

Model 1 was unadjusted.

Model 2 was adjusted for gender, age, race, education level, and RIP.

Model 3 was additionally adjusted for diabetes, hypertension, coronary heart disease, hypercholesterolemia, arthritis, stroke, cancer or malignancy, cigarette use, and alcohol use.

BRI = body roundness index, CI = confidence interval, OR = odds ratio, RIP = ratio of family income to poverty.

The tertile analysis showed a graded association. Compared with Q1, the fully adjusted common cumulative OR was 1.188 (95% CI, 1.055–1.337; *P* = .0064) for Q2 and 1.448 (95% CI, 1.272–1.647; *P* < .0001) for Q3. The trend across BRI tertiles was statistically significant in all 3 models (all *P* for trend < .0001).

### 3.4. Restricted cubic spline and threshold analyses

**Figure 2.**
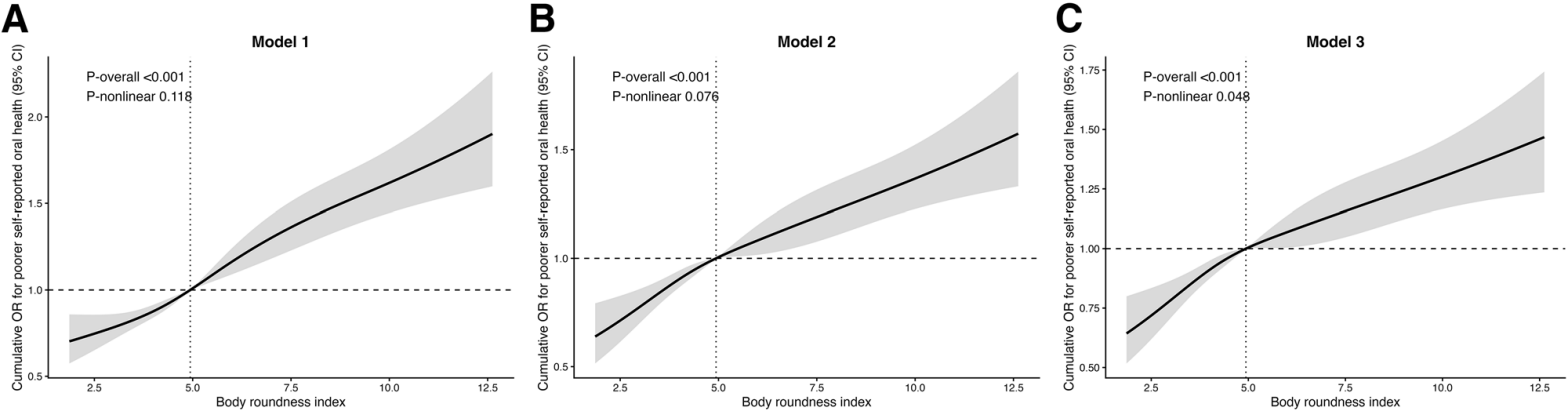
Dose–response association between body roundness index and poorer self-reported oral health. Restricted cubic spline models showing the association between BRI and poorer self-reported oral health. (A) Model 1 was unadjusted; (B) Model 2 was adjusted for gender, age, race, education level, and RIP; and (C) Model 3 was additionally adjusted for clinical and lifestyle covariates. BRI = body roundness index, CI = confidence interval, OR = odds ratio, RIP = ratio of family income to poverty.

Restricted cubic spline analyses showed significant overall associations between BRI and poorer self-reported oral health in Models 1 to 3 (all *P* for overall association < .001; Fig. 2). Evidence of nonlinearity was not statistically significant in Model 1 (*P* for nonlinearity = .118) or Model 2 (*P* for nonlinearity = .076), but reached statistical significance in Model 3 (*P* for nonlinearity = .048). The fully adjusted curve showed an overall increasing association, with attenuation at the upper end of the BRI distribution.

In the two-piecewise model with the folding point fixed at BRI = 10, the association was significant below the folding point. In Model 3, each 1-unit increase in BRI below 10 was associated with 9.2% higher common cumulative odds of poorer self-reported oral health (OR, 1.092; 95% CI, 1.063–1.120; *P* < .0001). At BRI values ≥10, the association was not statistically significant (OR, 0.978; 95% CI, 0.913–1.048; *P* = .5128). The ratio of the segment-specific ORs was 0.896 (95% CI, 0.822–0.977; *P* = .0147), indicating significant attenuation of the association above BRI = 10 (Table 4).

**Table 4:** Survey-weighted threshold analysis of the association between BRI and self-reported oral health.

Outcome: self-reported oral health	Model 2	Model 3
Linear-effect model		
Per 1-unit increase in BRI	1.085 (1.065, 1.106), <.0001	1.076 (1.055, 1.097), <.0001
Threshold-effect model		
Folding point (K)	10	10
BRI < 10	1.102 (1.074, 1.131), <.0001	1.092 (1.063, 1.120), <.0001
BRI ≥ 10	0.975 (0.914, 1.040), .4321	0.978 (0.913, 1.048), .5128
Ratio of segment ORs (≥ 10 vs <10)	0.885 (0.813, 0.962), .0054	0.896 (0.822, 0.977), .0147

Results are presented as cumulative OR (95% CI), *P*-value. OR > 1 indicates higher cumulative odds of being in a poorer self-reported oral-health category. The folding point was fixed at BRI = 10. The threshold model was compared with the linear model using a design-based Wald test of the hinge term.

Model 2 was adjusted for gender, age, race, education level, and RIP.

Model 3 was additionally adjusted for diabetes, hypertension, coronary heart disease, hypercholesterolemia, arthritis, stroke, cancer or malignancy, cigarette use, and alcohol use.

BRI = body roundness index, CI = confidence interval, OR = odds ratio, RIP = ratio of family income to poverty.

### 3.5. Assessment of the proportional-odds assumption

The proportional-odds assumption for BRI was not fully supported. The design-based Wald test of the threshold-by-BRI interaction was statistically significant in Model 1 (*P* = .019), Model 2 (*P* = .010), and Model 3 (*P* = .011). Nevertheless, the direction of the association was consistent across all 4 cumulative outcome thresholds (Table S3, Supplemental Digital Content 3). In Model 3, the threshold-specific ORs per 1-unit increase in BRI were 1.065 (95% CI, 1.025–1.106) for very good through poor versus excellent oral health, 1.077 (95% CI, 1.051–1.103) for good through poor versus excellent or very good oral health, 1.078 (95% CI, 1.052–1.104) for fair or poor versus excellent through good oral health, and 1.099 (95% CI, 1.066–1.132) for poor versus excellent through fair oral health. These findings indicate modest variation in effect magnitude across thresholds rather than a change in the direction of the association.

### 3.6. Subgroup analyses

**Figure 3.**
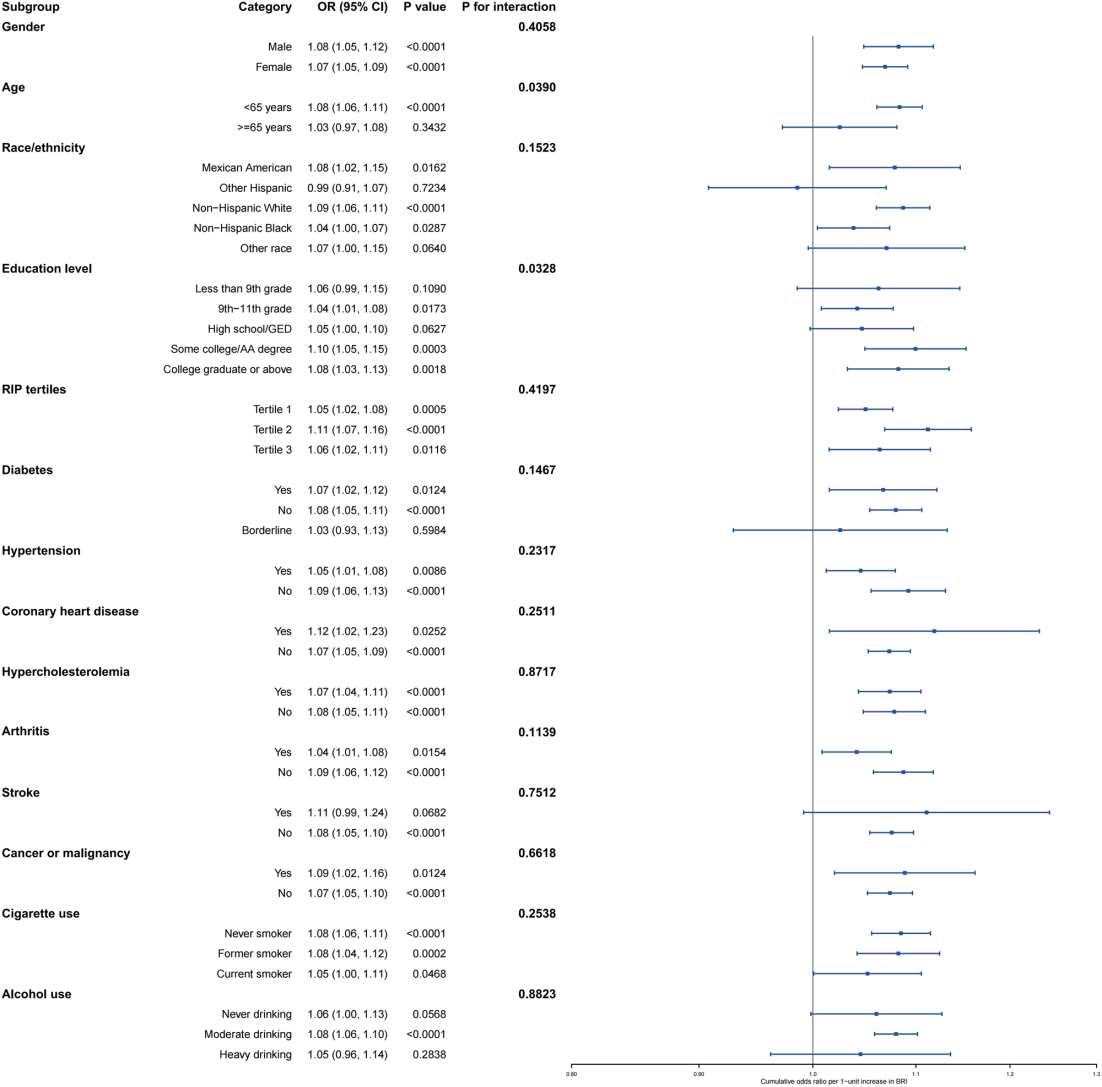
The results of subgroup analyses. CI = confidence interval, OR = odds ratio.

The positive association between BRI and poorer self-reported oral health was generally observed across demographic, socioeconomic, clinical, and lifestyle subgroups (Fig. 3). Statistically significant interactions were observed for age (*P* for interaction = .0390) and education level (*P* for interaction = .0328). No statistically significant interactions were observed for gender, race/ethnicity, RIP, diabetes, hypertension, coronary heart disease, hypercholesterolemia, arthritis, stroke, cancer or malignancy, cigarette use, or alcohol use (all *P* for interaction > .05). Stratified restricted cubic spline analyses are presented in Figure S1, Supplemental Digital Content 4.

### 3.7. Exploratory mediation analysis

The exploratory mediation analysis suggested that SII accounted for a small proportion of the statistical association between BRI and self-reported oral health (Table 5). After additional adjustment in Model 3, the estimated total effect was 0.034733 (95% CI, 0.026689–0.042900), the indirect effect was 0.001062 (95% CI, 0.000333–0.001931; *P* < .0001), and the direct effect was 0.033672 (95% CI, 0.025430–0.041869). The estimated proportion mediated was 3.06% (95% CI, 0.99%–5.82%; *P* < .0001). These estimates correspond to changes in the 1-to-5 oral-health score per 1-unit increase in BRI and indicate a small indirect statistical association through SII.

**Table 5:** Exploratory mediation analysis of SII in the association between BRI and self-reported oral health.

	Estimate	95% CI lower	95% CI upper	*P*-value
Model 2				
Total effect	0.042016	0.034329	0.049766	<.0001
Indirect effect	0.001631	0.000814	0.002563	<.0001
Direct effect	0.040385	0.032339	0.048367	<.0001
Proportion mediated, %	3.88	1.83	6.39	<.0001
Model 3				
Total effect	0.034733	0.026689	0.042900	<.0001
Indirect effect	0.001062	0.000333	0.001931	<.0001
Direct effect	0.033672	0.025430	0.041869	<.0001
Proportion mediated, %	3.06	0.99	5.82	<.0001

Outcome variable: self-reported oral health.

Mediator variable: SII.

Exposure variable: BRI.

Model 2 was adjusted for gender, age, race, education level, and RIP.

Model 3 was additionally adjusted for diabetes, hypertension, coronary heart disease, hypercholesterolemia, arthritis, stroke, cancer or malignancy, cigarette use, and alcohol use.

BRI = body roundness index, CI = confidence interval, RIP = ratio of family income to poverty, SII = systemic immune-inflammation index.

## 4. Discussion

In this nationally representative study of US adults, higher BRI was consistently associated with poorer self-reported oral health. This association remained significant after adjustment for demographic, socioeconomic, clinical, and lifestyle factors. In the fully adjusted model, each 1-unit increase in BRI was associated with a 7.6% increase in the cumulative odds of being in a poorer oral-health category, and participants in the highest BRI tertile had substantially higher odds than those in the lowest tertile. Restricted cubic spline and threshold analyses further suggested that the association was not entirely linear, while subgroup analyses indicated that the overall pattern was broadly consistent across most population groups.

BRI is an anthropometric indicator that combines WC and height and is considered a useful marker of central adiposity.^[28,29]^ Compared with general obesity, abdominal fat accumulation is more strongly related to insulin resistance, metabolic dysfunction, and chronic low-grade inflammation, all of which may adversely affect oral tissues.^[14,18,30]^ Excess adipose tissue can promote the release of proinflammatory mediators, impair immune regulation, and increase susceptibility to inflammatory responses in the gingiva and periodontal tissues.^[31]^ Obesity may also influence oral health indirectly through diabetes, hypertension, altered dietary patterns, reduced salivary function, and changes in the oral microbial environment.^[32,33]^ These interrelated metabolic and inflammatory pathways provide a biologically plausible explanation for the observed association between higher BRI and poorer self-reported oral health.

The threshold analysis provided additional information on the shape of the association. When BRI = 10 was used as the folding point, the association between BRI and poorer self-reported oral health was significant below this value but became weaker and statistically nonsignificant above it. This pattern suggests that increases in BRI may have a greater marginal influence within the lower-to-moderate range, whereas the association tends to plateau at very high BRI levels. One possible explanation is that, once severe central adiposity is established, metabolic abnormalities and chronic diseases may already be highly prevalent, reducing the additional independent contribution of further increases in BRI.^[34]^ Greater heterogeneity among individuals with very high BRI and the smaller number of observations in the upper range may also contribute to the flattening of the curve. Thus, the threshold pattern may reflect a combination of biological saturation and population distribution rather than a simple linear increase across the entire BRI range.^[24,35,36]^

The exploratory mediation analysis suggested that systemic inflammation may contribute to the association between BRI and self-reported oral health.^[37,38]^ SII showed a statistically significant indirect effect in both adjusted models, supporting the possibility that obesity-related immune and inflammatory activity is involved in the relationship.^[26,37,39]^ However, the estimated proportion mediated was small, decreasing from 3.88% in Model 2 to 3.06% in Model 3. Therefore, systemic inflammation appears to explain only a limited part of the overall association, and other metabolic, behavioral, and oral-health-related pathways are likely to play a larger role.^[40–42]^

This study has several strengths. It used a large, nationally representative sample and fully incorporated NHANES sampling weights, strata, and primary sampling units. The ordered nature of the oral-health outcome was addressed using survey-weighted ordinal logistic regression, and the consistency of the results across continuous, tertile-based, spline, threshold, and subgroup analyses supports the robustness of the findings.^[43,44]^ Nevertheless, several limitations should be acknowledged. The cross-sectional design prevents determination of temporal or causal relationships, and oral health was based on a single self-reported assessment rather than a clinical dental examination.^[45,46]^ Although common demographic factors, major chronic diseases, smoking, and alcohol use were included, other relevant factors such as oral-hygiene practices, dental attendance, access to dental care, diet, tooth loss, and denture use were not available or were not included.^[47]^ Residual confounding is therefore possible. Future longitudinal studies combining detailed oral-health behaviors with standardized clinical examinations are needed to confirm these findings and clarify the underlying mechanisms.

## 5. Conclusion

Higher BRI was significantly associated with poorer self-reported oral health among US adults, and this association persisted after adjustment for demographic, socioeconomic, clinical, and lifestyle factors. These findings support BRI as a potentially useful anthropometric indicator for identifying individuals at increased risk of poor perceived oral health. Further prospective studies incorporating clinical dental examinations and detailed oral-health–related factors are needed to confirm these findings and clarify the underlying mechanisms.

## Acknowledgments

We express our gratitude to the National Center for Health Statistics (NCHS) for granting access to the NHANES data and to all individuals who participated to this survey.

## Author contributions

**Conceptualization:** Wei Yang, Shuning Li.

**Funding acquisition:** Wei Yang, Shuning Li.

**Data curation:** Wei Yang, Shuning Li.

**Formal analysis:** Wei Yang, Shuning Li.

**Methodology:** Wei Yang, Shuning Li.

**Project administration:** Shuning Li.

**Validation:** Jilun Liu, Shuning Li.

**Visualization:** Jilun Liu, Shuning Li.

**Resources:** Shuning Li.

**Software:** Shuning Li.

**Investigation:** Wei Yang, Shuning Li.

**Supervision:** Shuning Li.

**Writing – original draft:** Jilun Liu, Shuning Li.

**Writing – review & editing:** Jilun Liu, Shuning Li.








