# Supplementary material for: Body roundness index and self-reported oral health among US adults: Nonlinear patterns and an exploratory indirect association through the systemic immune-inflammation index
Source: Medicine (Baltimore). 2026 Jul 24;105(30):e49981. doi: 10.1097/MD.0000000000049981 (PMC13406256; doi:10.1097/MD.0000000000049981)
Supplement: Supplementary file 1 [file medi-105-e49981-s001.docx]

**Table S1.** Calculation Methods and Classification Standards of Variables

|  | | Calculation methods or classification criteria |
| --- | --- | --- |
| Outcome Variable | |  |
| self-reported oral health |  |  |
|  | 1 | Excellent |
|  | 2 | Very good |
|  | 3 | Good |
|  | 4 | Fair |
|  | 5 | Poor |
| Exposure Variable |  |  |
| BRI | | $364.2-365.5\times\sqrt{1-\{\frac{(WC(cm)/{2\pi)}^{2}}{{(0.5\times height\left( cm \right))}^{2}}\}}$ |
| SII | | (Platelet count × Neutrophil count) / Lymphocyte count |
| Confounding Variables | |  |
| Cigarette Use |  |  |
| never smoker | | who have smoked fewer than 100 cigarettes in their lifetime |
| former smoker | | who have smoked more than 100 cigarettes but are currently abstinent |
| current smoker | | who have smoked more than 100 cigarettes and continue to smoke |
| Alcohol Use |  |  |
| never drinking | | who do not consume alcohol |
| moderate drinking | | characterized as men consuming 14 or fewer drinks per week or 4 or fewer drinks on any single day, and women consuming 7 or fewer drinks per week or 4 or fewer drinks on any single day |
| heavy drinking | | defined as men consuming more than 14 drinks per week or 5 or more drinks on any single day at least once in the past year, and women consuming more than 7 drinks per week or 5 or more drinks on any single day at least once in the past year |
