# Supplementary material for: Body roundness index and self-reported oral health among US adults: Nonlinear patterns and an exploratory indirect association through the systemic immune-inflammation index
Source: Medicine (Baltimore). 2026 Jul 24;105(30):e49981. doi: 10.1097/MD.0000000000049981 (PMC13406256; doi:10.1097/MD.0000000000049981)
Supplement: Supplementary file 2 [file medi-105-e49981-s002.docx]

**Table S2.** Unweighted baseline characteristics according to BRI tertiles

| Characteristic | Overall  N = 14,656^1^ | Q1  N = 4,634^1^ | Q2  N = 4,841^1^ | Q3  N = 5,181^1^ | p-value^2^ |
| --- | --- | --- | --- | --- | --- |
| Gender |  |  |  |  | <0.001 |
| male | 7,179 (49.0%) | 2,483 (53.6%) | 2,636 (54.5%) | 2,060 (39.8%) |  |
| female | 7,477 (51.0%) | 2,151 (46.4%) | 2,205 (45.5%) | 3,121 (60.2%) |  |
| Age group, years |  |  |  |  | <0.001 |
| <65 | 11,494 (78.4%) | 4,093 (88.3%) | 3,674 (75.9%) | 3,727 (71.9%) |  |
| ≥65 | 3,162 (21.6%) | 541 (11.7%) | 1,167 (24.1%) | 1,454 (28.1%) |  |
| Race |  |  |  |  | <0.001 |
| Mexican American | 2,020 (13.8%) | 371 (8.0%) | 758 (15.7%) | 891 (17.2%) |  |
| Other Hispanic | 1,468 (10.0%) | 380 (8.2%) | 518 (10.7%) | 570 (11.0%) |  |
| Non-Hispanic White | 6,266 (42.8%) | 1,981 (42.7%) | 2,044 (42.2%) | 2,241 (43.3%) |  |
| Non-Hispanic Black | 3,076 (21.0%) | 972 (21.0%) | 931 (19.2%) | 1,173 (22.6%) |  |
| Other Race | 1,826 (12.5%) | 930 (20.1%) | 590 (12.2%) | 306 (5.9%) |  |
| Education Level |  |  |  |  | <0.001 |
| Less Than 9th Grade | 1,363 (9.3%) | 216 (4.7%) | 491 (10.1%) | 656 (12.7%) |  |
| 9-11th Grade | 1,995 (13.6%) | 495 (10.7%) | 668 (13.8%) | 832 (16.1%) |  |
| High School Grad/GED or Equivalent | 3,347 (22.8%) | 996 (21.5%) | 1,091 (22.5%) | 1,260 (24.3%) |  |
| Some College or AA degree | 4,429 (30.2%) | 1,431 (30.9%) | 1,414 (29.2%) | 1,584 (30.6%) |  |
| College Graduate or above | 3,522 (24.0%) | 1,496 (32.3%) | 1,177 (24.3%) | 849 (16.4%) |  |
| RIP | 2.48 (1.60) | 2.62 (1.67) | 2.57 (1.61) | 2.27 (1.52) | <0.001 |
| Diabetes |  |  |  |  | <0.001 |
| YES | 1,751 (11.9%) | 159 (3.4%) | 487 (10.1%) | 1,105 (21.3%) |  |
| NO | 12,545 (85.6%) | 4,433 (95.7%) | 4,234 (87.5%) | 3,878 (74.9%) |  |
| Borderline | 360 (2.5%) | 42 (0.9%) | 120 (2.5%) | 198 (3.8%) |  |
| Hypertension |  |  |  |  | <0.001 |
| YES | 5,193 (35.4%) | 783 (16.9%) | 1,726 (35.7%) | 2,684 (51.8%) |  |
| NO | 9,463 (64.6%) | 3,851 (83.1%) | 3,115 (64.3%) | 2,497 (48.2%) |  |
| Coronary Heart Disease |  |  |  |  | <0.001 |
| YES | 546 (3.7%) | 79 (1.7%) | 194 (4.0%) | 273 (5.3%) |  |
| NO | 14,110 (96.3%) | 4,555 (98.3%) | 4,647 (96.0%) | 4,908 (94.7%) |  |
| Hypercholesterolemia |  |  |  |  | <0.001 |
| YES | 4,993 (34.1%) | 908 (19.6%) | 1,833 (37.9%) | 2,252 (43.5%) |  |
| NO | 9,663 (65.9%) | 3,726 (80.4%) | 3,008 (62.1%) | 2,929 (56.5%) |  |
| Arthritis |  |  |  |  | <0.001 |
| YES | 3,737 (25.5%) | 610 (13.2%) | 1,169 (24.1%) | 1,958 (37.8%) |  |
| NO | 10,919 (74.5%) | 4,024 (86.8%) | 3,672 (75.9%) | 3,223 (62.2%) |  |
| Stroke |  |  |  |  | <0.001 |
| YES | 465 (3.2%) | 89 (1.9%) | 141 (2.9%) | 235 (4.5%) |  |
| NO | 14,191 (96.8%) | 4,545 (98.1%) | 4,700 (97.1%) | 4,946 (95.5%) |  |
| Cancer Or Malignancy |  |  |  |  | <0.001 |
| YES | 1,307 (8.9%) | 299 (6.5%) | 436 (9.0%) | 572 (11.0%) |  |
| NO | 13,349 (91.1%) | 4,335 (93.5%) | 4,405 (91.0%) | 4,609 (89.0%) |  |
| Cigarette Use |  |  |  |  | <0.001 |
| never smoker | 8,164 (55.7%) | 2,666 (57.5%) | 2,671 (55.2%) | 2,827 (54.6%) |  |
| former smoker | 3,604 (24.6%) | 897 (19.4%) | 1,263 (26.1%) | 1,444 (27.9%) |  |
| current smoker | 2,888 (19.7%) | 1,071 (23.1%) | 907 (18.7%) | 910 (17.6%) |  |
| Alcohol Use |  |  |  |  | <0.001 |
| never drinking | 2,035 (13.9%) | 599 (12.9%) | 619 (12.8%) | 817 (15.8%) |  |
| moderate drinking | 12,135 (82.8%) | 3,880 (83.7%) | 4,060 (83.9%) | 4,195 (81.0%) |  |
| heavy drinking | 486 (3.3%) | 155 (3.3%) | 162 (3.3%) | 169 (3.3%) |  |
| Self-reported oral health |  |  |  |  | <0.001 |
| Excellent | 1,511 (10.3%) | 559 (12.1%) | 514 (10.6%) | 438 (8.5%) |  |
| Very good | 3,008 (20.5%) | 1,147 (24.8%) | 972 (20.1%) | 889 (17.2%) |  |
| Good | 5,392 (36.8%) | 1,703 (36.8%) | 1,791 (37.0%) | 1,898 (36.6%) |  |
| Fair | 3,213 (21.9%) | 863 (18.6%) | 1,084 (22.4%) | 1,266 (24.4%) |  |
| Poor | 1,532 (10.5%) | 362 (7.8%) | 480 (9.9%) | 690 (13.3%) |  |
| ^1^n (%); Mean (SD) | | | | | |
| ^2^Pearson's Chi-squared test; Kruskal-Wallis rank sum test | | | | | |
| Values are unweighted n (%) or mean (SD). P-values were calculated using the Pearson chi-square test or Kruskal–Wallis test. | | | | | |
