# Supplementary material for: Body roundness index and self-reported oral health among US adults: Nonlinear patterns and an exploratory indirect association through the systemic immune-inflammation index
Source: Medicine (Baltimore). 2026 Jul 24;105(30):e49981. doi: 10.1097/MD.0000000000049981 (PMC13406256; doi:10.1097/MD.0000000000049981)
Supplement: Supplementary file 3 [file medi-105-e49981-s003.docx]

**Table S3.** Survey-weighted threshold-specific associations between body roundness index and self-reported oral health and assessment of the proportional-odds assumption

| Model | Cumulative outcome contrast | OR (95% CI) | P value | P for threshold × BRI |
| --- | --- | --- | --- | --- |
| Model 1 | Very good–Poor vs Excellent | 1.077 (1.045–1.110) | <0.001 | 0.019 |
|  | Good–Poor vs Excellent/Very good | 1.111 (1.088–1.134) | <0.001 |  |
|  | Fair/Poor vs Excellent–Good | 1.112 (1.085–1.140) | <0.001 |  |
|  | Poor vs Excellent–Fair | 1.137 (1.106–1.168) | <0.001 |  |
| Model 2 | Very good–Poor vs Excellent | 1.061 (1.028–1.096) | <0.001 | 0.010 |
|  | Good–Poor vs Excellent/Very good | 1.085 (1.060–1.110) | <0.001 |  |
|  | Fair/Poor vs Excellent–Good | 1.087 (1.060–1.115) | <0.001 |  |
|  | Poor vs Excellent–Fair | 1.111 (1.082–1.141) | <0.001 |  |
| Model 3 | Very good–Poor vs Excellent | 1.065 (1.025–1.106) | 0.002 | 0.011 |
|  | Good–Poor vs Excellent/Very good | 1.077 (1.051–1.103) | <0.001 |  |
|  | Fair/Poor vs Excellent–Good | 1.078 (1.052–1.104) | <0.001 |  |
|  | Poor vs Excellent–Fair | 1.099 (1.066–1.132) | <0.001 |  |

Odds ratios represent the association with poorer self-reported oral health per 1-unit increase in BRI. Four survey-weighted binary logistic regression models were fitted at the cumulative outcome thresholds. P values for the proportional-odds assessment were obtained from design-based Wald tests of the threshold-by-BRI interaction. A statistically significant interaction indicates that the BRI coefficient differs across cumulative outcome thresholds. Model 1 was unadjusted; Model 2 was adjusted for gender, age, race/ethnicity, education level, and RIP; and Model 3 was additionally adjusted for diabetes, hypertension, coronary heart disease, hypercholesterolemia, arthritis, stroke, cancer or malignancy, cigarette use, and alcohol use.
