# Supplementary figures and images for: Body roundness index and self-reported oral health among US adults: Nonlinear patterns and an exploratory indirect association through the systemic immune-inflammation index
Source: Medicine (Baltimore). 2026 Jul 24;105(30):e49981. doi: 10.1097/MD.0000000000049981 (PMC13406256; doi:10.1097/MD.0000000000049981)

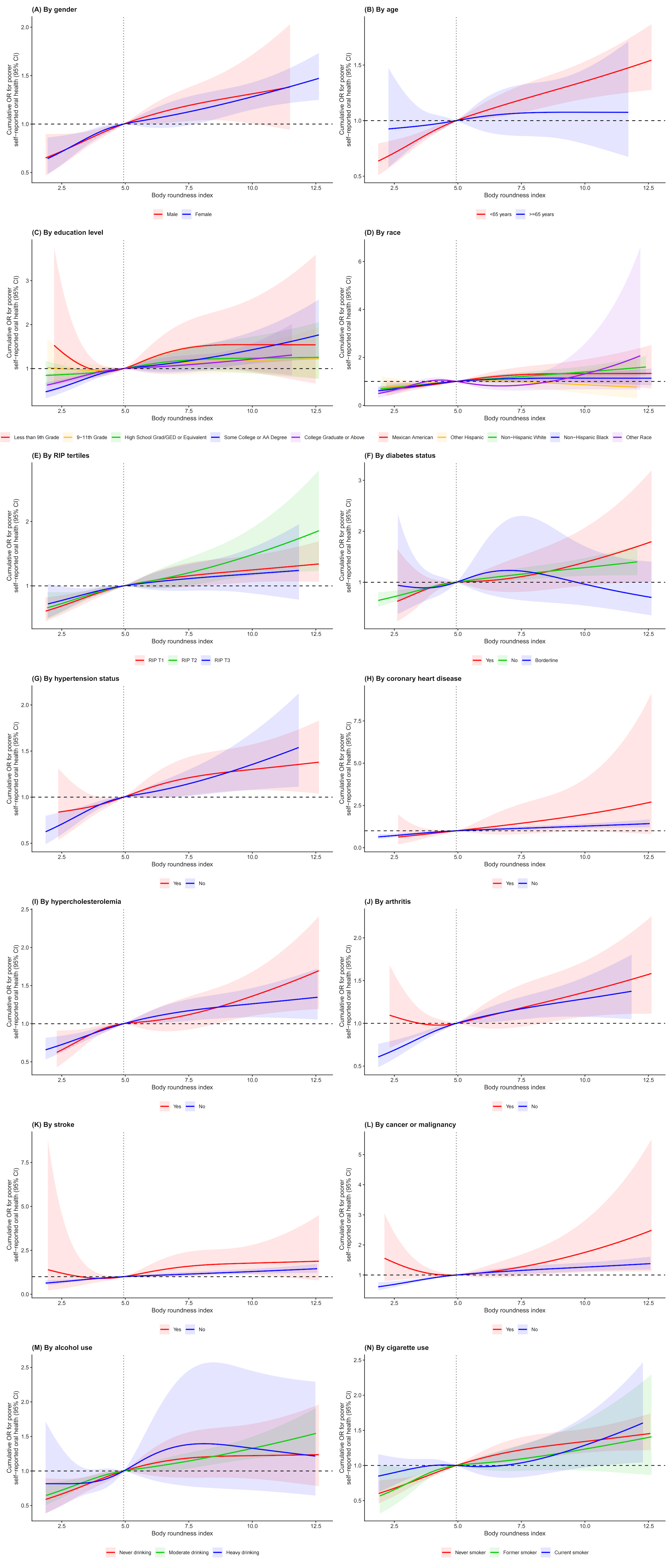

Supplement: Supplementary file 4 [file medi-105-e49981-s004.tiff]
